# Impact of the COVID-19 Pandemic on Elective and Emergency Inpatient Procedure Volumes in Switzerland – A Retrospective Study Based on Insurance Claims Data

**DOI:** 10.34172/ijhpm.2022.6932

**Published:** 2022-09-13

**Authors:** Yael Rachamin, Matthias R. Meyer, Thomas Rosemann, Thomas Grischott

**Affiliations:** ^1^Institute of Primary Care, University of Zurich and University Hospital Zurich, Zurich, Switzerland.; ^2^Division of Cardiology, Cantonal Hospital Graubuenden, Chur, Switzerland.

**Keywords:** COVID-19, Inpatient, Hospitals, Surgery, Undertreatment, Switzerland

## Abstract

**Background:** The severe acute respiratory syndrome coronavirus 2 (SARS‑CoV‑2) pandemic forced hospitals to redistribute resources for the treatment of patients with coronavirus disease 2019 (COVID-19), yet the impact on elective and emergency inpatient procedure volumes is unclear.

**Methods:** We analyzed anonymized data on 234 921 hospitalizations in 2017-2020 (55.9% elective) from a big Swiss health insurer. We used linear regression models to predict, based on pre-pandemic data, the expected weekly numbers of procedures in 2020 in the absence of a pandemic and compared these to the observed numbers in 2020. Compensation effects were investigated by discretely integrating the difference between the two numbers over time.

**Results:** During the first COVID-19 wave in spring 2020, elective procedure numbers decreased by 52.9% (95% confidence interval -64.5% to -42.5%), with cardiovascular and orthopedic elective procedure numbers specifically decreasing by 45.5% and 72.4%. Elective procedure numbers normalized during summer with some compensation of postponed procedures, leaving a deficit of -9.9% (-15.8% to -4.5%) for the whole year 2020. Emergency procedure numbers also decreased by 17.1% (-23.7% to -9.8%) during the first wave, but over the whole year 2020, net emergency procedure volumes were similar to control years.

**Conclusion:** Inpatient procedure volumes in Switzerland decreased considerably in the beginning of the pandemic but recovered quickly after the first wave. Still, there was a net deficit in procedures at the end of the year. Health system leaders must work to ensure that adequate access to non-COVID-19 related care is maintained during future pandemic phases in order to prevent negative health consequences.

## Background

**Key Messages**

** Implications for policy makers**

- The coronavirus disease 2019 (COVID-19) pandemic had a substantial impact on both elective and emergency procedures performed in Swiss hospitals over the year 2020.
- Policy-makers must work to ensure that adequate access to non-COVID-19 related care is maintained during future pandemic phases.
- A primary focus should be on investigating potential negative health consequences of postponed or cancelled procedures.

** Implications for the public**

In response to the coronavirus disease 2019 (COVID-19) pandemic, health systems were restructured to free up treatment capacity for patients with COVID-19 and to avoid in-hospital viral transmission. In the beginning of the pandemic, this involved cancellation of non-urgent surgeries. In this study, we estimated how many inpatient procedures were cancelled or postponed in Swiss hospitals in 2020 and how many were compensated until the end of the year. We found that inpatient procedure volumes decreased considerably in the beginning of the pandemic but recovered quickly after the first wave. Still, there was a net deficit in procedures at the end of the year. Our study sheds light on the impact of the pandemic on non-COVID-19 related care in Switzerland and underlines the importance of maintaining necessary treatment capacity. This could help policy-makers and clinicians to better respond to the next pandemic.

In response to the coronavirus disease 2019 (COVID-19) pandemic, healthcare systems were reorganized in order to free treatment capacity for patients with COVID-19 and to avoid in-hospital viral transmission.^1^ Consequently, decreases in the utilization of most non-COVID-19 related healthcare services have been reported, including ambulatory consultations, inpatient admissions and procedures, diagnostics, and therapeutics.^1-6^

 In Switzerland, non-urgent healthcare services were banned during the first wave of the pandemic in spring 2020.^7^ During that time, and in line with the international literature,^8-11^ Swiss physicians also reported seeing fewer patients for emergencies including stroke and myocardial infarction, which raised concerns of underuse of non-COVID-19 related healthcare. During the second wave in Switzerland starting in autumn 2020, COVID-19 infection- and hospitalization rates were much higher than in the first wave,^12^ and elective procedures were postponed again, although there was no nationwide ban on non-urgent treatments anymore.

 In Swiss primary care, reductions in consultation numbers during the first wave were relatively modest, and they were quickly back to normal after the ban was lifted.^13^ It is, however, conceivable that the impact on inpatient procedures was higher, since capping inpatient procedures was more critical in order to keep enough beds free for COVID-19 patients, and most hospital procedures could not be replaced by teleconsultations. To date, it is still unknown how the first and second wave impacted emergency and elective procedure volumes in Switzerland, and to what extent deficits during the first wave were compensated in the period between and after the two waves.

 Knowing how inpatient procedures were impacted by the pandemic and related countermeasures is crucial because a decrease in emergency cardiovascular or cancer-related procedures would support the frequent concerns of undertreatment of non-COVID-19 patients.^8-11,14-17^ Moreover, a lack of compensation of elective procedures including certain orthopedic surgeries may be an indication of a potential overtreatment in non-pandemic times.^3^ We therefore aimed to examine the impact of the COVID-19 pandemic on elective and emergency inpatient procedure volumes in the year 2020, with special focus on cardiovascular, orthopedic, and cancer-related procedures.

## Methods

###  Study Design and Setting

 We conducted a retrospective cohort study based on administrative claims data of a big Swiss health insurer in the years 2017-2019 (control) and 2020 (first year of the COVID-19 pandemic). No patient informed consent was required, as the study was retrospective and based on anonymized routine healthcare data (article 2 of the Swiss Federal Human Research Act^18^).

 In Switzerland, all residents are required to acquire mandatory health insurance.^19^ Residents can choose from over 50 health insurers and switch insurance contracts every year. The insured’s health insurer and canton of residence share inpatient treatment costs,^20^ which are reimbursed according to fixed-rate-per-case payment schedules that apply throughout Switzerland (Swiss Diagnosis Related Groups).^21^ We used data from Sanitas (Sanitas Grundversicherungen AG), which is the sixth-largest health insurer in Switzerland, providing mandatory basic health insurance to around 6% of Swiss residents (as of January 2020).^22^

###  Study Participants and Database Query

 We extracted data for all elective and emergency hospitalizations present in the claims database in 2017-2020 (calendar weeks 3-51; weeks 1-2 and 52 excluded due to sparse data). For each hospitalization, we extracted the year and calendar week of entry (as a proxy for the week when the procedure was conducted), the conducted procedures according to the Swiss surgical classification (CHOP),^23^ the main diagnosis according to the to the 10th revision of the International Classification of Diseases (ICD-10),^24^ and whether the patient was diagnosed with COVID-19. Furthermore, the following patient information was retrieved: Age (in age groups of 5 years), sex, number of diagnoses, and area (canton) of residence.

 Hospitalizations without any procedures were excluded from further analysis. In addition to overall elective and emergency procedure numbers, the following procedure groups were studied: cardiovascular procedures (CHOP chapter 7, elective and emergency), orthopedic procedures (CHOP chapter 14, elective and emergency; also including trauma surgical procedures), and cancer-related procedures (identified as procedures on patients who had the main diagnosis in ICD-10 chapter 2 ‘Neoplasms,’ because CHOP does not discriminate procedures in patients with or without cancer; emergency and elective were not separated, due to small sample size).

###  Definition of Pandemic Periods

 There were two COVID-19 waves in Switzerland in the year 2020, one in spring (March 16, 2020/calendar week 12 to April 26, 2020/calendar week 17), and one in autumn (from October 19, 2020/week 43, still ongoing at the end of the year 2020). During the first (but not the second) wave, there was a ban on non-urgent treatments, procedures and consultations.^7^ In general, Switzerland did not impose strict lockdowns (unlike other countries), but followed a strategy of gradual introduction and relief of disease containment measures instead.^25^ The strictness of these measures over time, in terms of the stringency index according to Pleninger et al,^26^ is illustrated in Figure S1 of Supplementary file 1, along with the two COVID-19 waves.

###  Objectives

 Objectives of this study were:

- To compare the weekly counts of elective and emergency procedures (overall, cardiovascular, orthopedic, and cancer-related procedures) in the pandemic year 2020 to expected numbers in the absence of a pandemic.
- To explore the net balances in procedure volumes at the end of the first wave and at the end of the year, and (potential) compensation effects over time.

###  Data Analysis

 We used counts (*n*) and proportions (%) or medians with interquartile ranges (IQRs) to describe the data. Groups were compared using the chi-square test and Kruskal-Wallis test, as appropriate.

 For each procedure group of interest (total 7 groups: elective overall, emergency overall, elective cardiovascular, emergency cardiovascular, elective orthopedic, emergency orthopedic, cancer-related), we counted the number of procedures for every week of every year. Multiple procedures within the same group were only counted once per hospital stay. We estimated weekly procedure numbers in the absence of a pandemic from linear regression models based on data from control years and the first 9 weeks of the year 2020 (week 9 was the week of the first diagnosed COVID-19 patient in Switzerland), with the independent variables ‘year’ (to account for differences in the insured population), ‘holidays’ (categorical variable; weeks with a public holiday and/or school holiday, see Table S1 of Supplementary file 1 for exact definitions), and a fifth degree polynomial of the standardized ‘calendar week’ (to account for seasonal differences; the degree of the polynomial was selected via Akaike information criterion). These expected (non-pandemic) procedure counts with bootstrapped pointwise 95% confidence bands were then plotted along with the actual observed values in 2020.

 To investigate compensation effects after the first wave, and to quantify net balances (deficits or surpluses) at the end of the first wave and the end of year with respect to the non-pandemic prediction, we discretely integrated the weekly differences between the observed and the expected numbers, starting at the beginning of the year. We then plotted these cumulative differences, again with bootstrapped pointwise 95% confidence bands, and reported net balances (both in absolute numbers and proportions with respect to cumulative counts of the non-pandemic prediction) with 95% confidence intervals (CIs) at the end of the first wave and at the end of the year.

 For overall elective and emergency procedures, the analyses were repeated *excluding* COVID-19 patients (to investigate non-COVID-19 related care). All analyses were carried out using the R software^27^ (Version 4.0.0).

## Results

###  Description of Cases

 We analyzed 234 921 hospitalizations with at least one performed procedure in the years 2017-2020. Of these hospitalizations, 176 702 (75.2%) were in control years (2017-2019) and 58 219 (24.8%) in the year 2020; 131 265 (55.9%) were elective and 103 656 (44.1%) were emergencies. Cardiovascular procedures were performed in 26 647 (11.3%), orthopedic procedures in 48 069 (20.5%) and cancer-related procedures in 24 329 (10.4%) hospitalizations. Characteristics of hospitalizations are given in Table.

**Table:** Description of Analysed Hospitalizations, Overall and by Calendar Year

	**Overall**	**Control Years**			**Pandemic Year**
	**2017–2020** **(n = 234 921)**	**2017** **(n = 58** **789)**	**2018** **(n = 58** **390)**	**2019** **(n = 59** **523)**	**2020** **(n = 58** **219)**
Elective, % (n)	55.9 (131 265)	58.5 (34 365)	56.5 (32 988)	55.1 (32 804)	53.4 (31 108)
Female patients, % (n)	54.2 (127 345)	54.5 (32 023)	54.0 (31 520)	54.4 (32 374)	54.0 (31 428)
Patient age (y), % (n)					
0-18	3.7 (8752)	3.7 (2152)	3.8 (2190)	4.0 (2393)	3.5 (2017)
19-50	25.8 (60 657)	26.5 (15 551)	25.2 (14 734)	25.7 (15 271)	25.9 (15 101)
51-80	53.0 (124 435)	53.5 (31 434)	53.7 (31 381)	52.4 (31 175)	52.3 (30 445)
81+	17.5 (41 077)	16.4 (9652)	17.3 (10 085)	17.9 (10 684)	18.3 (10 656)
Patient canton of residence, % (n)					
Zurich	33.7 (79 095)	33.4 (19 634)	33.8 (19 760)	33.7 (20 036)	33.8 (19 665)
Bern	11.1 (26 037)	10.8 (6375)	11.0 (6406)	11.0 (6555)	11.5 (6701)
Aargau	9.2 (21 548)	9.3 (5467)	9.0 (5250)	9.1 (5440)	9.3 (5391)
Basel-Country	6.0 (14 177)	6.1 (3563)	6.1 (3590)	5.9 (3524)	6.0 (3500)
Ticino	5.3 (12 354)	5.4 (3152)	5.3 (3103)	5.4 (3219)	4.9 (2880)
Thurgau	5.1 (12 018)	5.4 (3154)	5.2 (3039)	4.9 (2896)	5.0 (2929)
Others	29.7 (69 692)	29.7 (17 444)	29.5 (17 242)	30.0 (17 853)	29.5 (17 153)
Cases with COVID-19 diagnosis, % (n)	0.7 (1705)	0.0 (0)	0.0 (0)	0.0 (0)	2.9 (1705)
Number of diagnoses, median [IQR]	5 [3, 9]	5 [3, 8]	5 [3, 8]	5 [3, 9]	5 [3, 9]
With cardiovascular procedure/s, % (n)	11.3 (26 647)	11.7 (6879)	11.4 (6641)	11.4 (6764)	10.9 (6363)
With orthopedic procedure/s, % (n)	20.5 (48 069)	21.2 (12 435)	20.7 (12 078)	20.3 (12 074)	19.7 (11 482)
With cancer-related procedures, % (n)	10.4 (24 329)	10.2 (5983)	10.3 (6023)	10.5 (6227)	10.5 (6096)

Abbreviations: IQR, interquartile range; COVID-19, coronavirus disease 2019.

###  Overall Procedures

####  Elective Procedures

**Figure 1:**
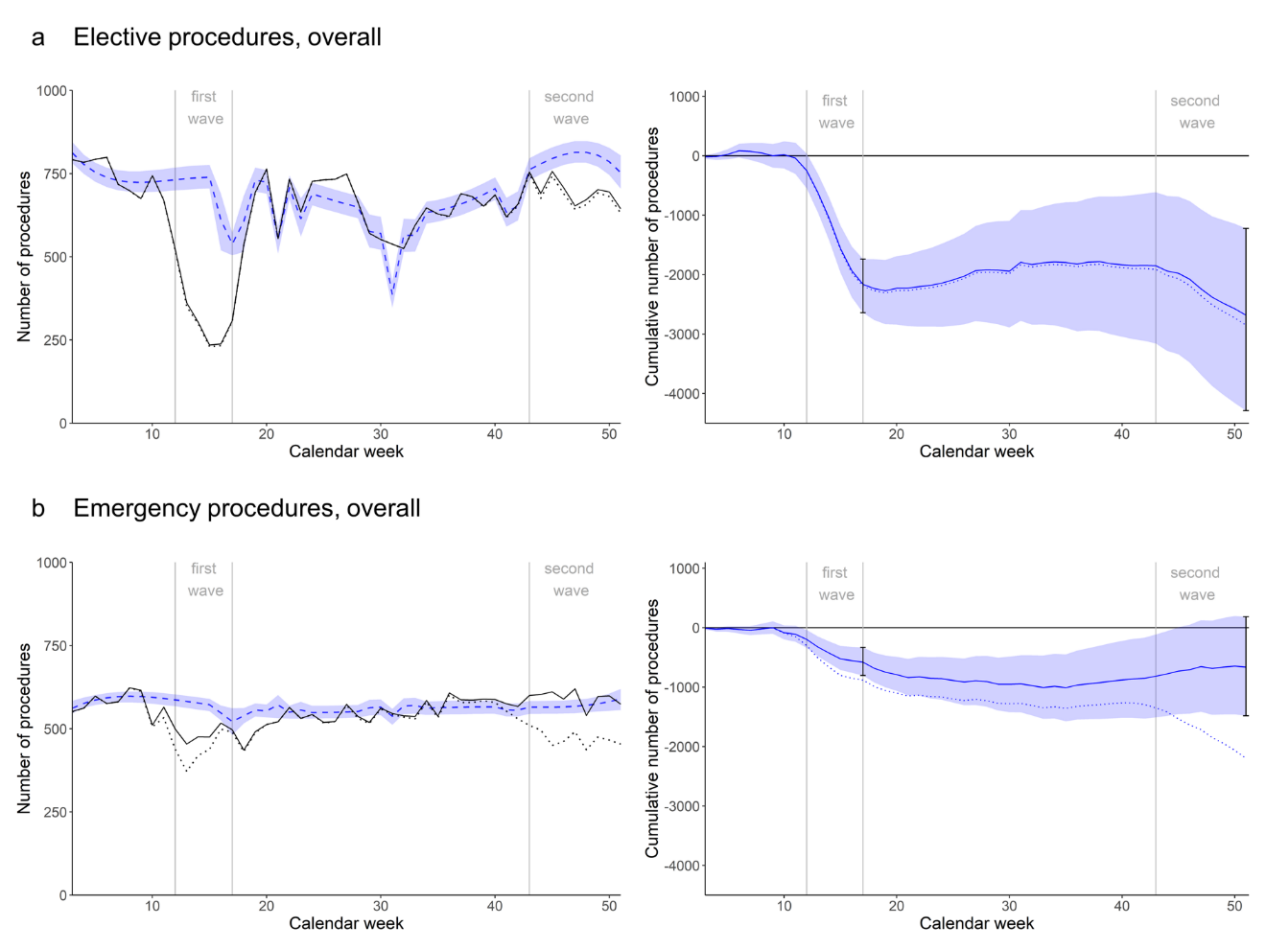
Total Elective (a, n = 31 108) and Emergency (b, n = 27 111) Procedures in 2020. Left panels: Predicted (blue dashed line, with pointwise 95% confidence bands) and observed weekly counts (black solid line: all patients; black dotted line: excluding COVID-19 patients). Right panels: Cumulative differences between predicted and observed weekly counts (blue solid line with pointwise 95% confidence bands: all patients; blue dotted line: excluding COVID-19 patients). 95% Confidence intervals of cumulative differences at the end of the first wave and at the end of the observation period are highlighted.

 During the first wave in spring 2020, elective procedure counts decreased, leaving a minus of -2164 procedures (95% CI -2642 to -1739) at the end of the first wave, which corresponds to a deficit of -52.9% (95% CI -64.5% to -42.5%) compared to the (non-pandemic) expectation (Figure 1a). There was a slight compensation in summer, before numbers decreased again during the second wave. At the end of the year 2020, the minus in elective procedures was -2682 (95% CI -4288 to -1223), representing a deficit of -9.9% (95% CI -15.8% to -4.5%) with respect to the expectation. Considering an average weekly elective procedure count of 690, the deficit in 2020 is equivalent to the number of procedures performed in -3.9 average non-pandemic weeks.

####  Emergency Procedures

 There was a minus of -579 (95% CI -804 to -331) emergency procedures in the first pandemic wave, corresponding to a deficit of -17.1% (95% CI -23.7% to -9.8%) compared to the expectation (Figure 1b). The deficit persisted for a few weeks after the end of the first wave, until the observed resumed to the expected procedure numbers. In the second wave, there was a slight increase due to procedures performed on patients with COVID-19: excluding patients with COVID-19 revealed that the reduction in emergency procedures during the second wave was similar to that during the first wave (Figure 1b). At the end of the year 2020, the minus in emergency procedures (including procedures on COVID-19 patients) was -662 (95% CI -1482 to 186), representing a deficit of -2.9% (95% CI -6.6% to 0.8%). Considering an average weekly emergency procedure count of 567, the deficit corresponds to around -1.2 average non-pandemic weeks.

###  Cardiovascular Procedures

####  Elective Procedures

**Figure 2:**
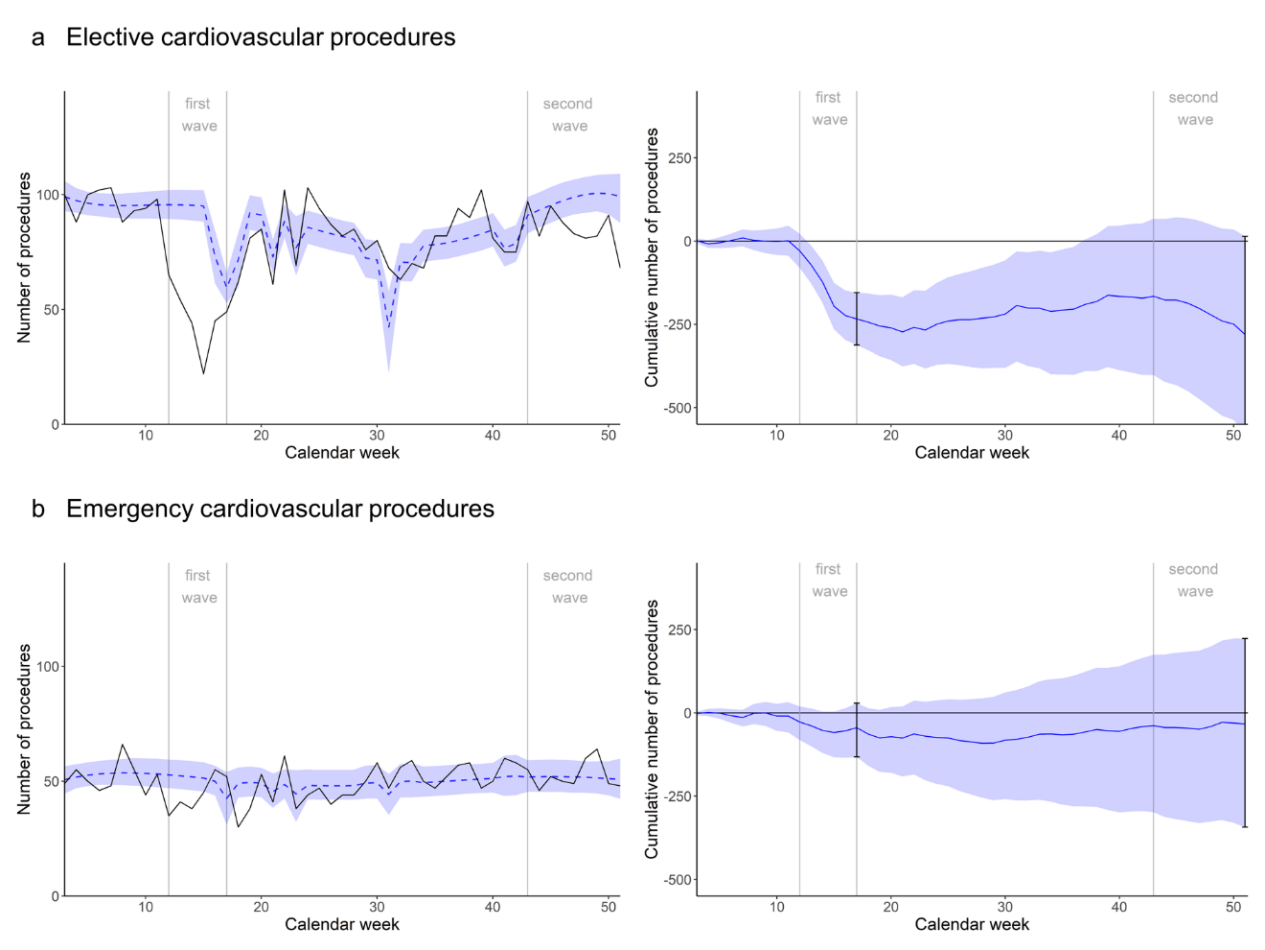
Cardiovascular Elective (a, n = 3929) and Emergency (b, n = 2434) Procedures in 2020. Left panels: Predicted (blue dashed line, with 95% confidence bands) and observed weekly counts (black solid line). Right panels: Cumulative differences between predicted and observed weekly counts (blue solid line with pointwise 95% confidence bands). 95% Confidence intervals of cumulative differences at the end of the first wave and at the end of the observation period are highlighted.

 Elective cardiovascular procedures were considerably lower than expected during the first wave, resulting in a minus of -234 (95% CI -312 to -155) at the end of the first wave, which corresponds to a deficit of -45.5% (95% CI -60.7% to -30.1%) compared to the expectation (Figure 2a). During the summer, there seemed to have been some compensation, before procedure counts moved below expectations again during the second wave. At the end of the year, the minus in cardiovascular elective procedures was -281 (95% CI -575 to 14), representing a deficit of -8.4% (95% CI -17.2% to 0.4%) compared to the expectation. Considering an average weekly elective cardiovascular procedure count of 85.9, the deficit corresponds to around -3.3 average non-pandemic weeks.

####  Emergency Procedures

 Emergency cardiovascular procedures also decreased slightly during the first wave, resulting in a minus of -45 procedures (95% CI -132 to 29), or -14.8% (95% CI -43.8% to 9.7%) at the end of the first wave (Figure 2b). They continued to be below expectation until midyear, but in the second half of the year, they seemed to have moved above expectation. Still, at the end of the year, the net deficit was -33 procedures (95% CI -343 to 223), which is a -1.7% deficit (95% CI -17.2% to 11.2%) of expected numbers in the period. Considering an average weekly emergency cardiovascular procedure count of 50, the deficit corresponds to around -0.7 average non-pandemic weeks.

###  Orthopedic Procedures

####  Elective Procedures

**Figure 3:**
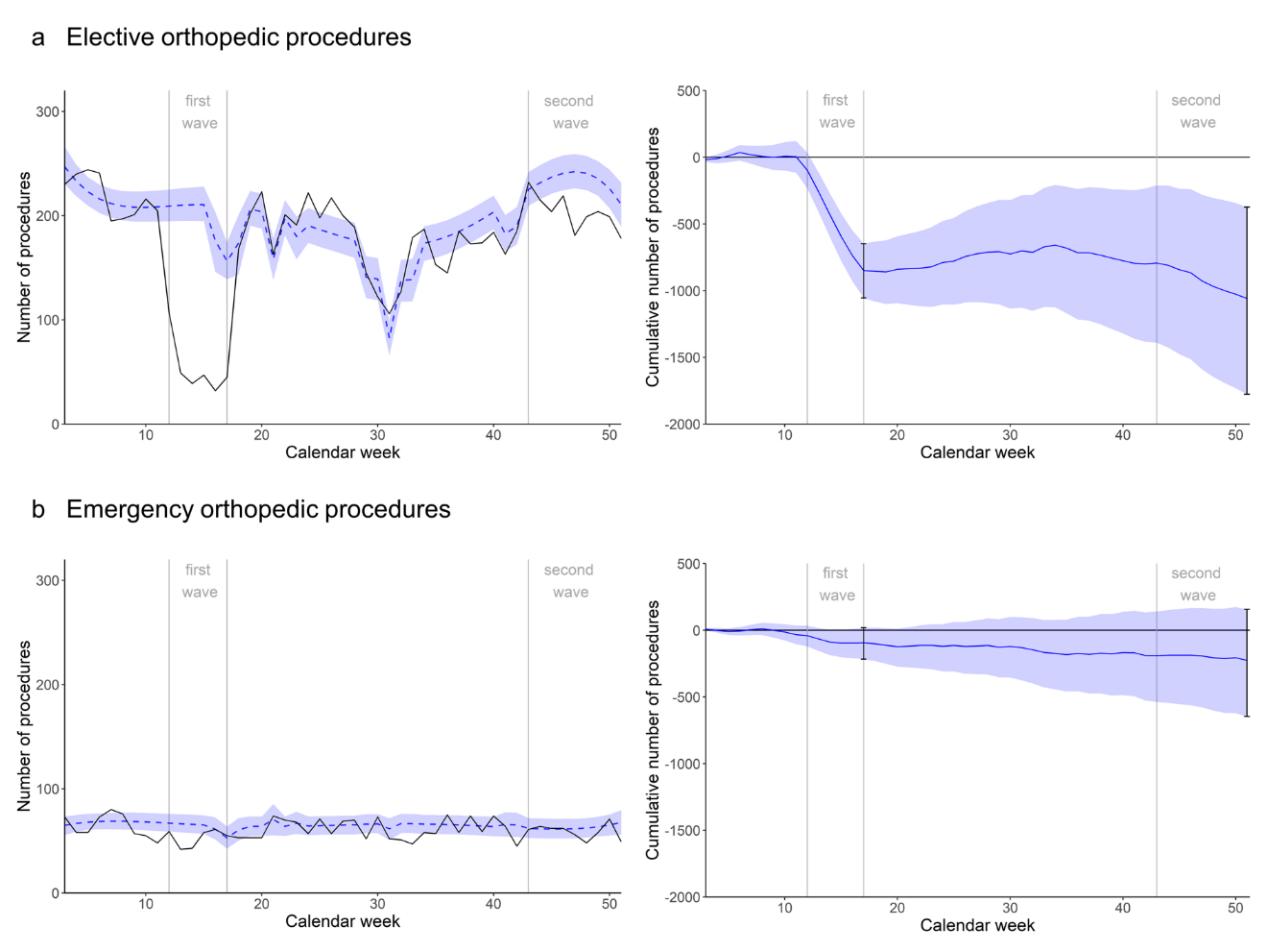
Orthopedic Elective (a, n = 8521) and Emergency (b, n = 2961) Procedures in 2020. Left panels: Predicted (blue dashed line, with pointwise 95% confidence bands) and observed weekly counts (black solid line). Right panels: Cumulative differences between predicted and observed weekly counts (blue solid line with pointwise 95% confidence bands). 95% Confidence intervals of cumulative differences at the end of the first wave and at the end of the observation period are highlighted.

 Elective orthopedic procedure volumes were considerably below expectation during the first wave (Figure 3a): At the end of the first wave, the minus was -849 (95% CI -1055 to -647), representing a -72.4% deficit (95% CI -90.0% to -55.2%). Until midyear, there was a slight compensation before procedure numbers started falling below expectation again, particularly during the second wave. At the end of the year, the minus in orthopedic elective procedures amounted to -1059 (95% CI -1776 to -373), representing a -13.9% deficit (95% CI -23.3% to -4.9%), corresponding to around -5.4 average non-pandemic weeks (of 196 elective orthopedic procedures per week).

####  Emergency Procedures

 Emergency orthopedic procedures were below expected values during the first and again during the second wave, with a short rebound to normality in summer (Figure 3b). At the end of the first wave, the minus amounted to -94 procedures (95% CI -217 to 19), or -24.8% (95% CI -57.2% to 5.0%), and at the end of the year, the deficit was -225 (95% -647 to 157), or -8.7% (95% CI -25.1% to 6.1%). This corresponds to around -3.5 average non-pandemic weeks (of 65 emergency orthopedic procedures per week).

###  Cancer-Related Procedures

**Figure 4:**
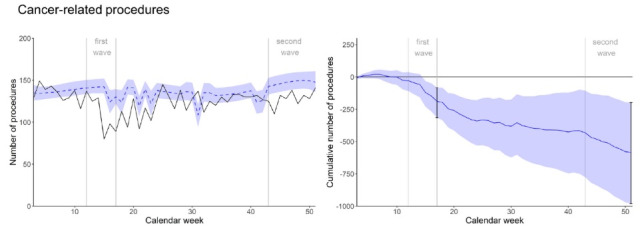
Cancer-Related Procedures in 2020 (n = 6096). Left panel: Predicted (blue dashed line, with pointwise 95% confidence bands) and observed weekly counts (black solid line). Right panel: Cumulative differences between predicted and observed weekly counts (blue solid line with pointwise 95% confidence bands). 95% Confidence intervals of cumulative differences at the end of the first wave and at the end of the observation period are highlighted.

 Procedure numbers of cancer patients were also below expectation in the first wave – with the minus at the end of first wave amounting to -188 procedures (95% CI -314 to -83), or -22.9% (95% CI -38.3% to -10.1%) – and remained considerably lower than expected for several weeks after the end of the first wave (Figure 4). While approaching expected numbers in late summer, they decreased again considerably in the second wave. At the end of the year, the minus in procedures was -584 (95% CI -981 to -199), or -10.7% (95% CI -18.0% to -3.6%), corresponding to -4.3 average non-pandemic weeks (of 136 cancer-related procedures per week).

## Discussion

 The two waves of the COVID-19 pandemic in 2020 had a significant, yet differential impact on elective and emergency procedures performed in Swiss hospitals. While elective procedures in 2020 were reduced by about 10% of volumes expected under non-pandemic conditions, the number of emergency procedures were below expected numbers during the first wave but comparable to non-pandemic years over the whole year. This can be explained by additional procedures required by patients with COVID-19, but also in part by an excess of emergency cardiovascular procedures over predicted volumes. Of note, a similar increase in emergency procedures in the second half of the year was not observed for orthopedic and cancer-related procedures.

 Substantial deficits in *elective* procedures during COVID-19 waves have previously been reported for different settings, overall^1,28^ as well as for distinct procedure groups including cardiovascular^29,30^ and orthopedic^31,32^ elective procedures. In Switzerland, a 43% decrease in elective visceral surgical procedures in a single tertiary hospital has been observed (during the first wave compared with the 6 weeks before).^33^ Interestingly, we found a quick and complete return of weekly elective procedure numbers to normal levels (not to be confused with a complete compensation) already in early summer, which contrasts studies from the United States^2^ and China^5^ reporting incomplete rebounds of inpatient services. This may be related to both differences in the pandemic burden and/or response, as well as differences in healthcare service capacities. Regarding *emergency* procedure volumes, the deficit we observed is also consistent with studies from different healthcare settings.^28,34-36^ In Switzerland, one study reported a 30% decrease in presentations to an emergency department during the first wave,^37^ while another reported about 40% fewer urgent visceral surgeries.^33^

 Most of the disruption in healthcare utilization may have been due to the reduction in service availability.^16^ However, particularly for urgent, non-deferrable healthcare services, patient-related factors might also have played a role, such as the patients’ fear of catching the virus at the hospital, concerns about adding an unnecessary burden on the healthcare system, and misperceptions of hospitals being open to COVID-19 patients only.^34,38-40^ Moreover, specifically for orthopedic emergency procedures, part of the decrease has been attributed to lifestyle changes resulting in fewer accidents.^34,41^

 Postponing or cancelling cardiovascular procedures has been of particular concern since the beginning of the pandemic, and a number of studies have since reported a negative impact on cardiovascular care.^8-11,14,15,29,30,42-47^ Importantly, several studies found declines in presentations and procedures for emergencies such as stroke and myocardial infarction.^8-11,15,42,44-49^ Recently, an analysis of a Swiss health insurer confirmed this finding, stating that mild myocardial infarctions decreased considerably during the first wave but also over the rest of the year 2020, compared to the year 2019.^50^ What at first glance seems contradictory to our findings of an increase in cardiovascular procedures during the second half of the year (Figure 2b) actually paints a more detailed picture of the situation: Contrary to our analysis, the authors of the previously mentioned study excluded myocardial infarctions which were treated by an interventional strategy (eg, percutaneous coronary intervention). Combined, the findings support the hypothesis that missed ‘mild’ emergencies, together with the cancelled or postponed (ambulatory and inpatient) elective treatments, were detrimental to the patients’ cardiovascular health, resulting in more emergency procedures later in the year. This is in line with a recent report on negative health outcomes of cardiovascular procedure postponing,^51^ and studies reporting increased out-of-hospital cardiovascular mortality rates during lockdowns.^46,52^

 Similar concerns as for cardiovascular diseases have been expressed for cancer, with studies reporting different observations: A study from Brazil found an increase in severe colorectal cancer presentations as a short-term effect of the pandemic,^53^ while in a European cohort, delaying radical prostatectomy for several months did not appear to adversely impact oncologic short-term outcomes.^54^ This may be in line with our observation that cancer-related procedures did not increase following the first wave, but whether this had detrimental effects on patients’ outcomes remains to be studied.

 Our results highlight the importance of maintaining healthcare for non-COVID-19 patients. It is currently unclear whether – or rather which – missed procedures had or will have a negative impact on patients’ health. Future research should investigate in more detail which procedures were missed, in order to determine which patients should be monitored closely and, in a second step, link missed procedures to potential negative health outcomes. This may also help to obtain a unique, more detailed overview on the perceived and actual importance of different procedures on patient outcomes. In addition to a closer look on different procedures, a detailed examination of patient subgroups is also warranted in order to identify particularly vulnerable patients for whom procedures should not be cancelled or postponed.^55^ Of note, for patients with severe acute respiratory syndrome coronavirus 2 (SARS-CoV-2) infection, postponement of surgery has actually been shown to be beneficial.^56^

 For elective procedures, it will also be interesting to investigate which specific procedures were compensated when and to what degree. Economic aspects might have played a role, as hospitals have suffered substantial financial losses.^57-59^ Interestingly, we observed some compensation of elective orthopedic procedures during summer months, which however stagnated several weeks before the beginning of the second wave, leaving open questions as to why. It is conceivable that resources, such as personnel and operation tables, were a limiting factor. Moreover, some of the cancelled procedures might not have been necessary after all (meaning they were indispensable in the first place). In this context, the COVID-19 pandemic has been described as an opportunity to reduce unnecessary healthcare.^3,60,61^

###  Strengths and Limitations

 Unlike most previous investigations of the impact of the COVID-19 pandemic on healthcare use, we described a period exceeding the first pandemic wave which allowed us to study potential compensation of cancelled or postponed procedures. Moreover, we chose a design and analysis that accounted for both temporal (year-to-year) and seasonal (within-year) differences.^4^

 Our study also has some limitations. First, our study is based on data derived from only one of over 50 health insurers, covering about 6% of the Swiss population and is not fully representative; for instance, the French-speaking part of Switzerland was underrepresented.^22^ Second, the sample was too small to discriminate different procedures within the investigated groups. Third, we did not account for the trend that a growing proportion of formerly inpatient procedures are shifted to the ambulatory setting in Switzerland.^62^ However, we do not expect that this significantly affected our analysis, since we included the first few weeks of 2020 in the prediction to control for such non-pandemic time trends. Lastly, it should be noted that some of the deficit in procedure volumes is attributable to people who died from COVID-19. Assuming 420 deaths from COVID-19 in our sample (6% of the 7000 confirmed deaths from COVID-19 in Switzerland in 2020^12^), this would, in a first approximation, correspond to a maximum of 13% of the minus of 3344 (emergency and elective) procedures that we observed.

## Conclusion

 We observed considerable pandemic-related declines in elective procedure volumes that were not compensated until the end of 2020, and a decrease in most emergency procedures. An exception were cardiovascular emergency procedures, which seemed to have increased in the second half of the year 2020, raising concerns of negative health consequences from cancelled or postponed procedures during earlier stages of the pandemic. Our results suggest that health system leaders should not lose sight of providing access to needed non-COVID-19 related care during future pandemic phases, particularly for cardiovascular diseases.

## Acknowledgements

 We would like to thank Pascal Godet from Sanitas health insurance for providing the data for the present study and Levy Jäger and Fabio Valeri from the University of Zurich for statistical consulting.

## Ethical issues

 No patient informed consent was required, as the study was retrospective and based on anonymized routine health care data (article 2 of the Swiss Federal Human Research Act).

## Competing interests

 TR reports honoraria from Novartis, Amgen, BMS, Grünenthal, Mepha, Daiichi Sankyo, and Boehringer Ingelheim. YR, MRM and TG declare that they have no conflict of interest.

## Authors’ contributions

 YR: conceptualization, formal analysis, funding acquisition, validation, writing–original draft, writing–review & editing. MM: conceptualization, writing–review & editing; TR: conceptualization, resources, writing–review and editing; TG: conceptualization, methodology, validation, writing–original draft, writing– review & editing.

## Funding

 This research was partly funded by the Federal Office of Public Health and by Novartis. The funders had no influence on study design, data analysis, preparation of the manuscript, or decision to publish.

## Supplementary files

Supplementary file 1 contains Table S1 and Figure S1.
