# Supplementary material for: Impact of the COVID-19 Pandemic on Elective and Emergency Inpatient Procedure Volumes in Switzerland – A Retrospective Study Based on Insurance Claims Data
Source: Int J Health Policy Manag. 2022 Sep 13;12:6932. doi: 10.34172/ijhpm.2022.6932 (PMC10125178; doi:10.34172/ijhpm.2022.6932)
Supplement: Supplementary file 1 — contains Table S1 and Figure S1. [file ijhpm-12-6932-s001.pdf]

Article title: Impact of the COVID-19 Pandemic on Elective and Emergency Inpatient Procedure Volumes in Switzerland – A Retrospective Study Based on Insurance Claims Data

Journal name: International Journal of Health Policy and Management (IJHPM)

Authors' information: Yael Rachamin[1]*, Matthias R. Meyer[2,1], Thomas Rosemann[1], Thomas Grischott[1]

[1]Institute of Primary Care, University of Zurich and University Hospital Zurich, Zurich, Switzerland.

[2]Division of Cardiology, Cantonal Hospital Graubuenden, Chur, Switzerland.

(*Corresponding author: yael.rachamin@usz.ch)

Supplementary file 1

Table of content:

Figure S1, p. 2
Table S1, p. 3

Figure S1. Timeline of Swiss COVID-19 measures stringency in 2020. The different lines represent different cantons of Switzerland, with the red line indicating the canton of Zurich, which was the most represented canton in the study.

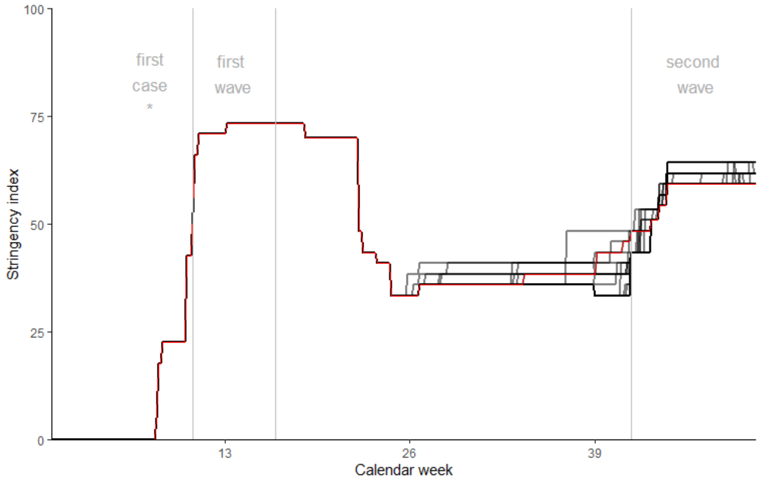

Table S1. Definition of the holiday variable, for each calendar week of the years 2017-2020. We extrapolated school holidays from the canton of Zurich, which represents 30% of the data, to the whole sample. Each distinct holiday (e.g., 'Christmas holiday', 'Easter holiday, 'summer holiday) was treated as a separate category. If a holiday period exceeded two subsequent weeks, the central week/s was/were treated as an additional, distinct category (e.g., 'Midsummer holiday'). 'Sports holidays' (typically in February) were disregarded because their time point varies greatly between cantons.

Calendar week	Holiday Variable			
	Year 2017	Year 2018	Year 2019	Year 2020
1[a]	-	-	-	-
2[a]	-	-	-	-
3	Ref.	Ref.	Ref.	Ref.
4	Ref.	Ref.	Ref.	Ref.
5	Ref.	Ref.	Ref.	Ref.
6	Ref.	Ref.	Ref.	Ref.
7	Ref.	Ref.	Ref.	Ref.
8	Ref.	Ref.	Ref.	Ref.
9	Ref.	Ref.	Ref.	Ref.
10	Ref.	Ref.	Ref.	Ref.
11	Ref.	Ref.	Ref.	Ref.
12	Ref.	Ref.	Ref.	Ref.
13	Ref.	Easter holidays	Ref.	Ref.
14	Ref.	Easter holidays	Ref.	Ref.
15	Spring holidays	Ref.	Ref.	Ref.
16	Midspring holidays	Ref.	Spring holidays	Spring holidays
17	Spring holidays	Spring holidays	Midspring holidays	Midspring holidays
18	Ref.	Spring holidays	Spring holidays	Spring holidays
19	Ref.	Ascension holiday	Ref.	Ref.
20	Ref.	Ref.	Ref.	Ref.
21	Ascension holiday	Whitsun holiday	Ref.	Ascension holiday
22	Ref.	Ref.	Ascension holiday	Ref.
23	Whitsun holiday	Ref.	Ref.	Whitsun holiday
24	Ref.	Ref.	Whitsun holiday	Ref.
25	Ref.	Ref.	Ref.	Ref.
26	Ref.	Ref.	Ref.	Ref.
27	Ref.	Ref.	Ref.	Ref.
28	Ref.	Ref.	Ref.	Ref.
29	Summer holidays	Summer holidays	Summer holidays	Summer holidays
30	Summer holidays	Summer holidays	Summer holidays	Summer holidays
31	Midsummer holidays	Midsummer holidays	Midsummer holidays	Midsummer holidays
32	Summer holidays	Summer holidays	Summer holidays	Summer holidays

33	Summer holidays	Summer holidays	Summer holidays	Summer holidays
34	Ref.	Ref.	Ref.	Ref.
35	Ref.	Ref.	Ref.	Ref.
36	Ref.	Ref.	Ref.	Ref.
37	Ref.	Ref.	Ref.	Ref.
38	Ref.	Ref.	Ref.	Ref.
39	Ref.	Ref.	Ref.	Ref.
40	Ref.	Ref.	Ref.	Ref.
41	Autumn holidays	Autumn holidays	Autumn holidays	Autumn holidays
42	Autumn holidays	Autumn holidays	Autumn holidays	Autumn holidays
43	Ref.	Ref.	Ref.	Ref.
44	Ref.	Ref.	Ref.	Ref.
45	Ref.	Ref.	Ref.	Ref.
46	Ref.	Ref.	Ref.	Ref.
47	Ref.	Ref.	Ref.	Ref.
48	Ref.	Ref.	Ref.	Ref.
49	Ref.	Ref.	Ref.	Ref.
50	Ref.	Ref.	Ref.	Ref.
51	Ref.	Ref.	Ref.	Ref.
52[a]	-	-	-	-

[a] Calendar week one, two, and 52 were not analyzed, see Methods section.
Abbreviations: Ref., Reference/no holiday
